# Chiral communication in polymer systems: generation and regulation of asymmetric structures

**DOI:** 10.1039/d6sc01021k

**Published:** 2026-04-14

**Authors:** Zixiang He, Gong Zhang, Wei Zhang

**Affiliations:** a College of Chemistry, Zhengzhou University Zhengzhou 450001 China zixianghe@zzu.edu.cn weizhang@zzu.edu.cn; b School of Material Science and Engineering, Henan University of Technology Zhengzhou 450001 China

## Abstract

Chiral communication represents a fundamental characteristic of living systems and serves as a critical bridge linking molecular chirality with the macroscopic properties of chiral materials. In chiral polymer systems, chiral information can be effectively transmitted across different building units, spatial scales, and hierarchical levels through covalent and noncovalent interactions. This process allows subtle chiral biases to be progressively accumulated and amplified into coincident chiral conformations, which can be further expressed in supramolecular assemblies and even macroscopic materials. A profound understanding and precise regulation of chiral communication in polymer systems not only provides a significant theoretical foundation for the rational design of novel chiral functional materials but also helps reveal the intrinsic laws governing the origin and transmission of chirality in living systems. In this perspective, we present a concise overview of chiral communication in polymer systems and elucidate its underlying mechanisms. Representative examples are highlighted to illustrate the generation and regulation of asymmetric structures driven by chiral communication, along with an overview of related applications in chiral functional materials, which aims to offer new insights to transcend existing research paradigms and propel the further development of this field.

## Abstract 2

This perspective summarizes chiral communication in polymer systems and offers insights for the design of advanced chiral functional materials.

## Graphical abstract

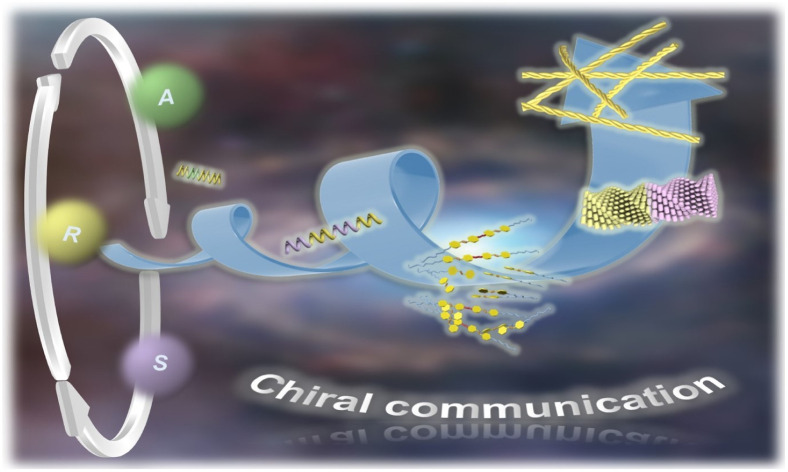

## Introduction

Chirality is a ubiquitous form of asymmetry intrinsically linked to the origin of life.^1^ Within biological systems, chiral symmetry and selectivity are fundamental to biological functionality.^2^ The homochirality of biological macromolecules is crucial for life, as their complex functions are often determined by higher-order topological chiral structures that emerge from individual homochiral building units. The transmission of chiral information from lower-level chirality (*e.g.*, molecular point chirality) to higher-order chiral architectures (*e.g.*, macroscopic chiral structures) relies critically on chiral communication among these homochiral units, a process that profoundly influences biological activities. Consequently, researchers have been devoted to constructing precisely ordered chiral functional materials through the selective synthesis and supramolecular assembly of chiral small molecules, biological macromolecules, and helical polymers, aiming to mimic and explore fascinating phenomena in living systems.^3,4^

In recent years, synthetic helical polymers have rapidly become a major focus in chemistry and materials science.^5,6^ Notably, constructing chiroptical polymers with specific helical preferences based on chiral communication offers an efficient and versatile strategy. Chiral communication in polymer systems typically follows a multi-level pathway, propagating from molecular to supramolecular scales and from the microscopic to the macroscopic level.^7^ Since the ground-breaking concept of “supramolecular chemistry” was introduced by Lehn in 1978 based on host-guest chemistry, the field of supramolecular chirality in polymers has seen significant breakthroughs.^8,9,10,11^

**Fig. 1:**
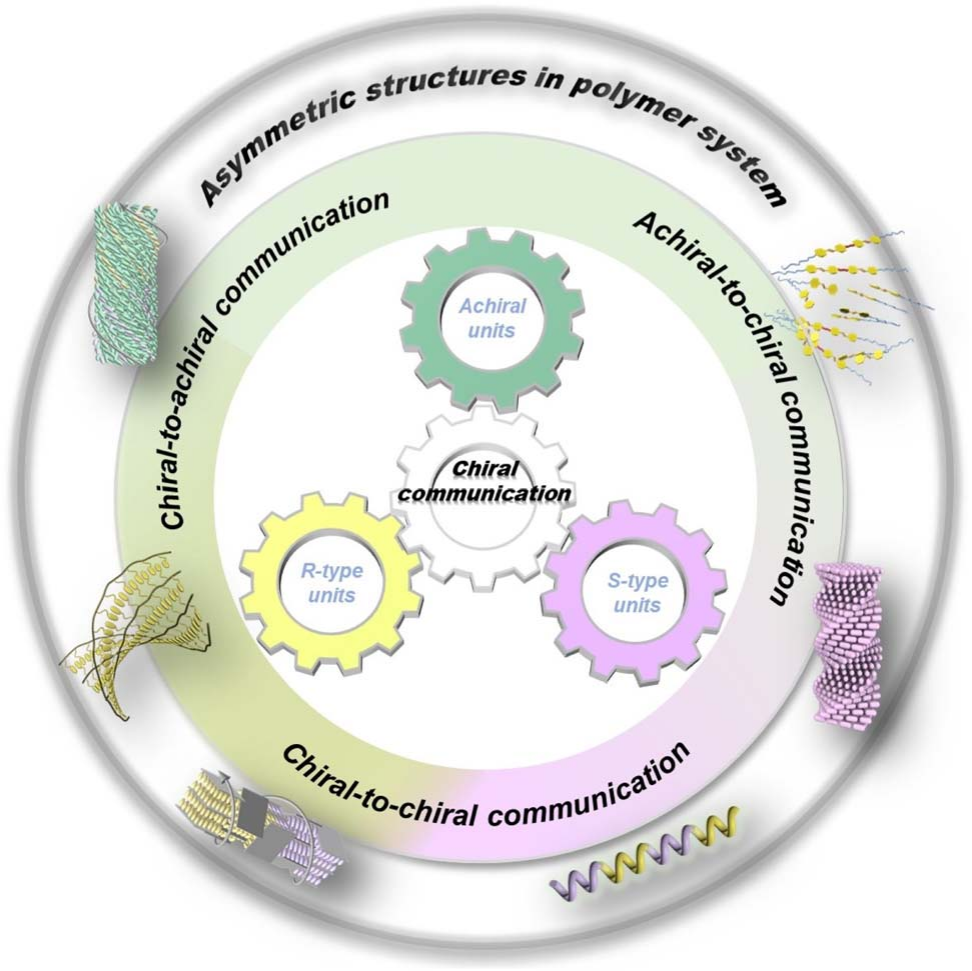
Schematic representation of asymmetric amplification originating from chiral communication between different basic building units, including both enantiomeric (*R*-type and *S*-type) and achiral components.

Supramolecular chirality in polymer systems refers to chiral, ordered structures where the polymer backbone or its internal building units spontaneously adopt a dominant helical conformation through noncovalent interactions such as hydrogen bonding, van der Waals forces, hydrophobic effects, and π–π stacking.^11–14^ This chirality does not originate from the intrinsic chirality of monomeric units in chiral polymers but emerges from cooperative intermolecular interactions, resulting in an overall chiral arrangement at higher hierarchical levels (*e.g.*, nanoscale or microscale). In contrast to small molecule systems, supramolecular chiral polymers that are covalently linked combine the structural stability of covalent bonds with the versatility of noncovalent interactions.^15^ They feature tunable secondary helical structures and multiple assembly pathways, thereby offering promising application prospects in chiroptical materials, asymmetric catalysis, and biomaterials.^16,17^ The rapid advances in supramolecular chemistry in recent years have further enabled the precise construction and dynamic regulation of internal asymmetric helical structures in polymer systems through different chiral communication between tailored building units, including both chiral and achiral components (Fig. 1).

In this perspective, we focus on the mechanisms of asymmetric amplification and chiral transfer induced by chiral communication, with particular emphasis on delineating the modes of chiral communication in polymer systems and their regulatory effects on helical asymmetric structures. Using representative examples, we highlight the pivotal role of chiral communication in the construction, regulation, and functionalization of helical architectures, which is essential for advancing the practical applications of chiral polymers. Furthermore, we discuss the advantages and limitations of current approaches and identify key unresolved challenges and open questions in the study of chiral communication within chiroptical polymer systems.

### Different chiral communication strategies in chiroptical polymer systems

With the rapid development of chiroptical polymer materials, a wide variety of artificially synthesized helical polymers have been reported to date, including poly(methyl methacrylate)s, polyisocyanides, polysilanes, polyacetylenes, polyisocyanates, polyaldehydes, polystyrenes, polyguanidines, and conjugated polymers.^18–21^ Chiral communication is ubiquitous within helical polymer systems, accompanied by a unique and particularly fascinating phenomenon known as asymmetric amplification.^22^ Helical structures can be induced into a single-handed helical conformation by covalent or noncovalent chiral stimuli, whereby an extremely small screw sense bias along the polymer chain is dramatically amplified, resulting in enhanced optical activity or enantiomeric excess (ee). Fundamentally, this phenomenon arises from the formation of chiral secondary helical conformations along the polymer backbone. Since the pioneering discovery by Green and co-workers in 1989,^23^ extensive mechanistic studies have progressively unveiled multiple pathways for asymmetric amplification.^24,25^

Based on the distinct components involved in chiral communication, this phenomenon, arising from interactions between adjacent building units and subsequent conformational communication, can be broadly categorized into three types: (i) chiral communication between enantiomeric building units with opposite stereocenters; (ii) chiral communication between a small fraction of chiral units and a large number of achiral units; and (iii) chiral communication between two distinct types of chiral building units. With the rapid evolution of chiral communication mechanisms, this field is no longer confined to polymers but has also been extensively studied in supramolecular self-assembly systems.

**Fig. 2:**
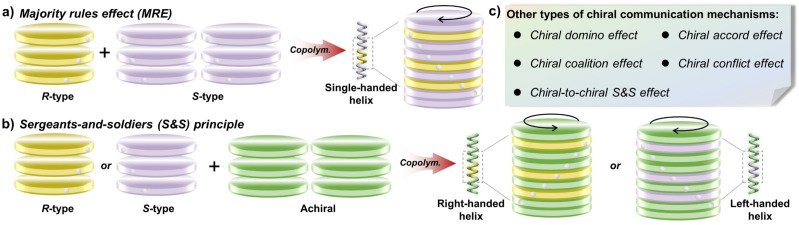
Different chiral communication mechanisms in chiroptical polymer systems. (a) Chiral communication between enantiomeric building units (majority rules effect, MRE). (b) Chiral communication between chiral and achiral building units (sergeants-and-soldiers, S&S principle). (c) Other types of chiral communication mechanisms derived from the MRE and S&S principles.

Chiral communication between enantiomeric building units: in systems containing mixtures of enantiomers with opposite configurations, the copolymerization or co-assembly of non-racemic building units generates the asymmetric arrangement that exhibits a helical sense preference disproportionate to its ee value. Specifically, a slight excess of one enantiomer strongly biases the system toward the helical sense preferred by that enantiomer. This results in a non-linear dependence between optical activity (typically circular dichroism, CD signals) and the ee value. Consequently, a helical system constructed from two enantiomeric comonomers in a ratio different from 1 : 1 can exhibit a helical excess comparable to that observed in systems derived from homochiral monomer units. This phenomenon, known as the “majority rules effect” (MRE),^26,27^ is a quintessential asymmetry amplification effect and holds significant implications for understanding the origin of homochirality in nature (Fig. 2a).

Chiral communication between chiral and achiral building units: in self-assembled systems composed of chiral and achiral building units, the regulation of the helical structure can be achieved through a primary chiral transfer mechanism. Here, chiral units (the minority “sergeants”, *R*- or *S*-type) induce a specific asymmetric arrangement in the achiral building units (the majority “soldiers”).^28^ This asymmetry amplification mechanism is termed the “sergeants-and-soldiers” (S&S) principle,^29^ whereby a minute fraction of chiral building units dominates the overall helical secondary structure of the assembly by directing the screw sense excess of achiral components (Fig. 2b). Furthermore, studies on chiral–achiral communication and chiral induction have revealed that covalently attaching chiral groups to the termini of achiral polymer chains can also induce single-handed helical conformations under specific conditions (*e.g.*, temperature and solvent). This terminal chiral induction transfer mechanism generates an intrinsic helical sense preference and enhances preferred conformation, thereby enabling asymmetric amplification, referred to as the “chiral domino effect”.^30^

Chiral communication between heterochiral building units: beyond the effects described above, differential expression arising from binary combinations of chiral sergeants and chiral soldiers in dynamic chiral polymers enables selective control over polymer helicity. In dynamic helical polymers with low helix inversion energy barriers, interactions between chiral sergeants and chiral soldiers have likewise been shown to regulate helical structures and induce asymmetric amplification. In these systems, in addition to the helical bias induced by side chains along the polymer backbone, other communication mechanisms between different components in copolymers must also be considered. Through systematic investigations of chiral communication between binary units with distinct chiral structures, a series of chiral communication mechanisms have been proposed, including the “abnormal S&S effect”,^31^ “chiral-to-chiral S&S effect”,^32^ “chiral coalition effect”,^33^ “chiral accord effects”^34^ and “chiral conflict effects”^35^ (Fig. 2c). These heterochiral communication mechanisms, derived from the MRE and S&S principles, are currently focused primarily on main-chain helical polymer systems and will be illustrated through specific case studies in subsequent sections.

Overall, chiral communication and asymmetric amplification in polymer and supramolecular systems can be achieved through interactions between enantiomeric building units, chiral–achiral building units, or heterochiral building units. These strategies provide simple and effective approaches for the precise construction and regulation of chiral superhelical architectures, thereby facilitating their further functional exploration and practical applications of chiroptical polymers.

### Generation and regulation of asymmetric structures *via* chiral communication

Artificial systems exhibiting chiral communication enable uniform conformational ordering of chiral components, amplifying a global chiral bias and offering compelling prospects for chemistry and materials. Most reported artificial systems with chiral communication capabilities employ static, non-adjustable structures aimed at tight chiral coupling, which may limit their processability and chirality tunability. In contrast, the synergistic interplay of covalent bonds and weak noncovalent interactions in polymers offers the potential to concurrently achieve both dynamic behavior and structural stability. In this section, we will explore the construction and regulation of polymer systems endowed with helicity control over chiral communication.

#### Chiral communication within main-chain helical polymers

Classical asymmetric amplification arising from chiral communication was first discovered in main-chain helical polyisocyanates characterized by low helix inversion energy barriers.^23,24^ Both the MRE and the S&S principles originate from the strong cooperative interactions among isocyanate units along the polymer backbone, whereby a small chiral bias in chiral isocyanate units is dramatically amplified into a pronounced main-chain helicity. Importantly, both effects are based on fully dissolved polymer systems, in which the accumulation of small local energetic preferences along the helical backbone ultimately results in an excess of a specific helical sense across the entire polymer backbone. Subsequent studies demonstrated that these two effects are not limited to polyisocyanates but are also widespread and have been extensively investigated in other classes of main-chain helical polymers, including polysilanes, polyacetylenes, polyquinoxalines, poly(phenylacetylene)s, and so on.^4,36–39^

**Fig. 3:**
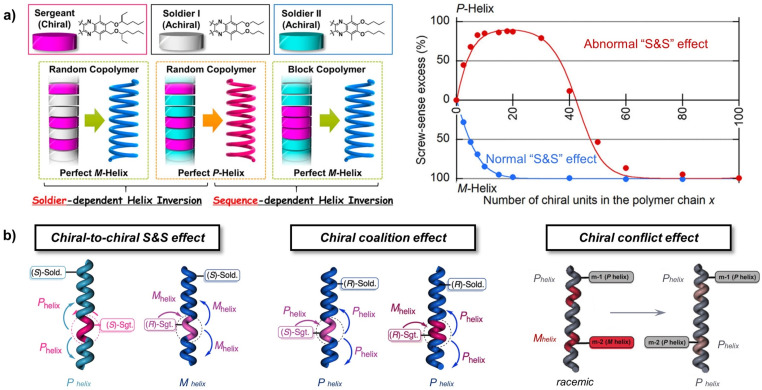
(a) Reversal of the main-chain helicity triggered by the copolymerization strategy and the abnormal S&S effect in the polyquinoxaline system; reproduced from ref. 31. Copyright 2015, The American Chemical Society. (b) Chiral-to-chiral S&S effect, chiral coalition effect, and chiral conflict effect in the main-chain helical polymer systems; reproduced from ref. 34. Copyright 2024, The American Chemical Society.

With the continued advancement of chiral polymer research, an unusual type of asymmetric amplification that does not conform to the conventional S&S principle was identified and termed the “abnormal S&S effect”.^40^ In this case, a gradual increase in the content of sergeant units induces a reversal of the overall helical sense of the polymer backbone. Suginome and co-workers first reported this abnormal S&S effect in polyquinoxaline systems, where the helical sense of the polymer backbone is dictated by the fraction of sergeant units incorporated into the polymer (Fig. 3a).^31^ Moreover, even when the molar fraction of sergeant monomers is kept constant, switching the copolymerization strategy, from random to block copolymerization, can also trigger a reversal of the main-chain helicity. These observations indicate that polymer helicity can be regulated not only by chiral composition but also by the copolymerization sequence, which effectively modulates the distribution of chiral sergeants and achiral soldiers. The abnormal S&S effect can be well rationalized using the Sato model (a modified Ising model).^29^

Notably, conventional helical copolymer systems typically exhibit only a single S&S mode, either normal or abnormal. In contrast, Wan *et al.* reported a unique conformation-switchable S&S effect in helical random poly(phenylacetylene) copolymers, in which solvent polarity modulates hydrogen-bonding interactions to precisely control the transition between contracted and extended helical conformations.^41^ In the contracted helical state, variations in the chiral unit content induce a reversal of helical sense, corresponding to an abnormal S&S effect, whereas in the extended conformation, optical activity increases monotonically with increasing chiral component content, characteristic of a normal S&S effect. Recent studies further demonstrate that, beyond solvent effects, dynamic regulation of asymmetric amplification within a single system can also be achieved through metal-ion coordination, phase-structure effects, and related external stimuli.

In heterochiral helical polymers, chiral communication most commonly proceeds through a mechanism in which chiral sergeant monomers exert specific control over the configuration of chiral soldier units, a process that is closely associated with changes in the absolute configuration of the chiral soldier units. A representative example is the chiral-to-chiral S&S effect,^32^ which was systematically investigated by Freire *et al.* using phenylacetylene-based chiral monomers (Fig. 3b). Although the soldier monomers possess stereocenters on the pendant benzene rings, the polymer exhibits an axial racemic state (an equal coexistence of *P*- and *M*-type helical conformations) in the absence of external stimuli. This phenomenon arises from an equilibrium between two pendant group conformations: anti-periplanar (*ap*) and *syn*-periplanar (*sp*). These distinct conformations result in different spatial arrangements of the chiral groups, thereby inducing opposite helical senses in the polymer backbone. However, when the pendant benzene ring is replaced with an anthracene ring, this conformational equilibrium is disrupted, shifting from the *sp* to the *ap* conformation. Consequently, the chiral soldier units align into a specific helical conformation under the induction of the chiral sergeants. This transition renders the originally axially racemic polymer optically active, resulting in asymmetry amplification.

In addition, Green and co-workers reported another distinct pathway of chiral communication, termed the “chiral conflict effect”,^42^ which was identified in early studies of copolymers composed of two distinct chiral components. In these systems, the two heterochiral monomers are individually capable of inducing opposite helical senses, leading to a competition between the opposing helical conformations (Fig. 3b).^35^ Investigating helically tunable chiral transfer mechanisms within the chiral dyad component copolymers is important for the precise construction of macromolecular chirality and its practical applications. For example, Zou *et al.* reported an asymmetry amplification effect with tunable characteristics in chiroptical poly(phenylacetylene) copolymers.^43^ In an enantiomeric copolymer system using chloroform as the solvent, the helical sense excess of the dissolved polymer chain was found to be dominated by the minority chiral enantiomer. Conversely, when the solvent was switched to tetrahydrofuran, the system exhibited a chiral conflict behaviour, with the preferred helical sense being completely opposite to that observed in chloroform. By exploiting this controllable asymmetric amplification mechanism, they successfully constructed an intelligent helical regulation system capable of performing Boolean logic operations, thereby providing new insights for the development of smart polymer materials based on controllable helical architectures, with potential applications in optical information storage, chiral sensing, and related fields.

#### Chiral communication in main-chain chiroptical polymer assemblies

The above discussion has focused on the regulation of polymer helical conformations through chiral communication between the basic building units, while chiral polymer aggregates, which have emerged as a major research focus on supramolecular chemistry, have also attracted increasing attention with respect to their chiral communication behaviours. It is well established that, in the absence of any external asymmetrical intervention, racemic polymer systems composed of equimolar enantiomeric building units are inherently difficult to achieve deracemization.^44^ This difficulty arises from the strong covalent bonding between enantiomeric units, which severely limits effective molecular rearrangement, and from the inability of racemic supramolecular assemblies to undergo controllable, selective assembly. Consequently, deracemization into homochiral polymer aggregates is rarely achieved under such conditions.

**Fig. 4:**
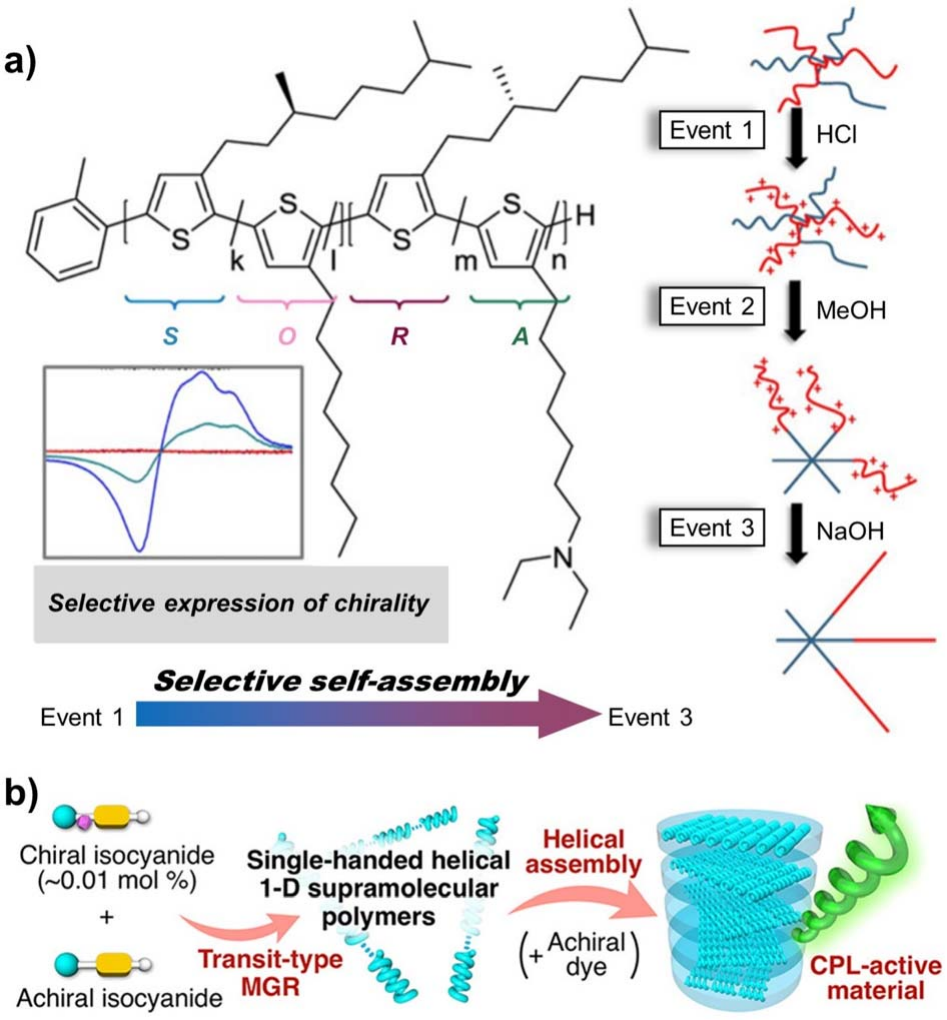
(a) Post-polymerization self-assembly and selective expression of dominant helicity in the polythiophene *via* adjusting the assembly conditions; reproduced from ref. 46. Copyright 2015, The American Chemical Society. (b) Helical assembly of single-handed helical supramolecular polymers and hierarchical chiral transfer in the liquid crystalline (LC) film; reproduced from ref. 50. Copyright 2020, The American Chemical Society.

Block copolymers, composed of segments with distinct aggregation characteristics, represent ideal model systems for investigating the aggregation behaviour of chiral polymers.^45^ Typically, upon the addition of a poor solvent, the block with lower solubility aggregates preferentially. This initial aggregation pattern subsequently dictates the assembly behaviour of the remaining block, such that the overall aggregation process follows a “first-come, first-served” (FF) principle. As a result, supramolecular self-assembly of block copolymers provides an effective strategy for inducing chiroptical activity even in copolymers composed of equimolar enantiomeric building units.^46–49^ As shown in Fig. 4a, one segment of the copolymer (*S* + *O*) consists of an *S*-configured chiral block and the achiral block *O*, while the other segment (*R* + *A*) comprises an equal number of *R*-configured chiral blocks and the achiral block *A* in the polythiophene. During post-polymerization self-assembly in solution, regulation of the assembly conditions can induce pronounced differences in the solubility of the *R*- and *S*-blocks, thereby triggering chiral conflict that ultimately leads to the selective expression of a dominant helicity. In this context, the relative solubility of different blocks determines the aggregation priority and, to a large extent, governs the packing mode of the copolymer. In addition, terminal groups, achiral side-chain substituents, and the degree of polymerization (DP) all exert significant influences on aggregation behaviour.^46^ Therefore, supramolecular chiral self-assembly and chiral expression generally require specifically designed end-group structures to break inversion symmetry and enable the emergence of chiral signals.

Moreover, in the construction of asymmetric structures, chiral polymers can achieve both weak interaction-induced supramolecular helical stacking and covalent bond-mediated helical sense transmission and overpass. These lead to the formation of supramolecular assemblies with well-defined helicity, enabling the efficient transfer of chiral information and the hierarchical amplification of asymmetric structures. For example, Ikai and co-workers reported the copolymerization of benzodithiophene-appended achiral/chiral isocyanates (molar ratio = 99 : 1) with solid-state photoluminescent materials, leading to the formation of submicrometer supramolecular fibers (Fig. 4b).^50^ In these assemblies, nearly perfect single-handed helical polyisocyanate segments are connected through noncovalent interactions resulting in asymmetric amplification. The resulting helical supramolecular polymers further undergo hierarchical chiral self-assembly to form cholesteric liquid crystalline (LC) films. Moreover, the chiral information can be efficiently transferred to achiral fluorescent dyes, enabling full-colour and tunable induced circularly polarized luminescence (CPL). Therefore, the structural and functional design of helical polymers at the molecular scale is of profound significance. It not only helps elucidate the interaction mechanisms between local asymmetric structures and global helical superstructures during assembly but also enables the exploration of the fundamental mechanism governing chiral communication and transfer within main-chain helical polymer systems, ultimately advancing the functional application of chiral polymer materials.

#### Chiral communication in hierarchical chiral assembly of side-chain chiral polymers

Traditional research on the chirality of main-chain helical polymers has primarily focused on the molecular level within polymer backbones, with limited exploration into the hierarchical transfer from molecular to supramolecular scales. Beyond these rigid systems, another extensively studied class of chiral polymers comprises flexible backbones bearing side-chain substituents.^51,52^

In these systems, helicity typically arises from the asymmetric supramolecular stacking of side-chain building units. A representative example is the azobenzene (Azo) containing LC polymer system.^53–55^ In such polymers, chiroptically active assemblies can be constructed through a combination of asymmetric polymerization strategies and supramolecular chiral self-assembly, thereby enabling chiral transfer across multiple levels and asymmetric amplification. As discussed earlier in the context of the chiral domino effect, our group synthesized side-chain polymers with chiral Azo building units at the α-terminus. Through solution-based post-polymerization self-assembly, the chiral information was successfully transferred from the terminal chiral units to the achiral Azo segments, resulting in an amplification of the asymmetry effect that yielded the well-defined supramolecular asymmetric structures.^56^ This strategy, predicated on chiral communication between a single chiral building unit and numerous achiral building units, significantly reduces the reliance on costly chiral sources and provides a simple yet effective strategy for constructing tunable and functional chiral polymer and supramolecular architectures.

In the fabrication of chiral polymer assemblies, post-polymerization assembly and polymerization-induced chiral self-assembly (PICSA) represent two major approaches.^57^ The former typically involves polymer synthesis followed by self-assembly, as exemplified by conventional solution self-assembly methods. This strategy is often hampered by limitations such as being time-consuming, involving tedious procedures, and having low efficiency (with solid content typically below 1 wt%). Furthermore, controlling and monitoring chiral communication during post-polymerization assembly is challenging, often precluding *in situ* mechanistic studies. In contrast, the PICSA strategy, which has advanced rapidly since 2020, allows for the precise construction and large-scale preparation of hierarchical supramolecular chiral structures *in situ* during the polymerization process, opening new avenues for controllable fabrication and hierarchical chiral evolution of polymer assemblies. In PICSA, the communication between building units influences asymmetric packing at the molecular level and helical suprastructures at the supramolecular level, leading to asymmetry amplification.^58,59^ Simultaneously, the chiral communication between building units and the assembly modes of PICSA are mutually influential. In current PICSA research, the investigation of amplification of asymmetry triggered by chiral communication is still in an early exploratory stage.

**Fig. 5:**
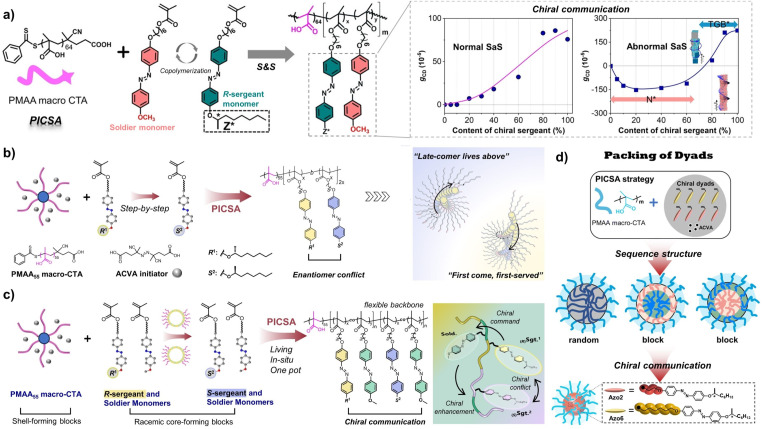
(a) Unconventional commands between chiral sergeant units and achiral soldier units and different chiral communication mechanisms in the LC-S&S system *via* the PICSA strategy; reproduced from ref. 60. Copyright 2023, The American Chemical Society. (b) Step-wise PICSA of enantiomeric Azo units and the different chiral conflict effects. (c) Chiral communication between enantiomeric and achiral monomers; reproduced from ref. 62. Copyright 2025, WILEY-VCH Verlag GmbH & Co. KGaA. (d) Chiral-to-chiral communication between dyads with distinct stacking tendencies; reproduced from ref. 63. Copyright 2023, The Royal Society of Chemistry.

In 2023, our group reported a study on switching the supramolecular chirality of nucleation segments in the LC PICSA system *via* unconventional commands between chiral sergeant units and various achiral soldier units.^60^ As illustrated in Fig. 5a, during the process of hierarchical chiral transfer and asymmetric amplification, supramolecular chirality dominated the chiroptical activity of the resulting polymer assemblies. Notably, this asymmetric amplification was independent of the absolute configuration of the chiral sergeant units and exhibited a synergistic effect between normal S&S behaviour (in the amorphous state) and abnormal S&S behaviour (in the LC phase). The DP of the solvophobic block, the liquid crystallinity of the achiral soldier segments, and the mole fraction of the chiral sergeant units collectively dictate the efficiency of asymmetry amplification. Chiral communication between building units in these LC-S&S systems likely results in distinct energy barriers. Accordingly, the precise regulation of the supramolecular helical conformation can be achieved by increasing the DP of the nucleation block, adjusting the sergeant-to-soldier ratio, or modifying the liquid crystallinity of the achiral units, which provides a novel phase-transition model for tuning the helicity of chiral polymer architectures within PICSA systems.

In addition, we also reported chiral communication between enantiomeric building units. By utilizing step-wise PICSA of enantiomeric Azo units, global supramolecular chirality and dynamic helicity switching were realized in racemic polymer systems without any external asymmetrical intervention (Fig. 5b).^61^ The transition of Azo enantiomeric units from an amorphous phase (intra-chain asymmetric arrangement) to a LC phase (inter-chain *H*-aggregation), alongside the intrinsic stability of the LC “chiral core”, drives multiple inversions of chiroptical activity during living polymerization, self-assembly, and chiral conflict. Specifically, the dynamic switching between left- and right-handed supramolecular chirality can be regulated by controlling the competition between the “FF” and “late-comer lives above” (LA) effects. This controllable construction of homochiral supramolecular structures from equimolar enantiomers offers a viable strategy for preparing chiroptical materials without external asymmetric intervention.

In addition to enantiomeric building units, achiral compounds, which are abundant in nature, play a critical role in chiral communication and asymmetry amplification. Recently, a new chiral communication mode based on interactions between enantiomeric and achiral monomers was proposed (Fig. 5c).^62^ In this system, equimolar Azo mesogens with opposite chirality act as sergeant units that trigger chiral communication processes (including chiral coalition and chiral conflict) within the solvophobic LC core. This induces the achiral Azo soldiers to form asymmetric supramolecular structures, facilitating the formation of global chirality and multiple helical inversions within racemic nano-assemblies.

Furthermore, the living polymerization characteristics of PICSA provide a versatile platform for various combinations of building units. When different structural Azo dyads are introduced into the PICSA system, chiral building units with distinct stacking tendencies facilitate the communication and transfer of chiral information during supramolecular self-assembly (Fig. 5d).^63^ The relative ratio of chiral comonomers, the molecular weight of the solvophobic block, and variations in polymer sequence structures collectively enable tunable chiral-to-chiral communication. Importantly, chiroptical activities in these systems are not dictated by stereocenters of the basic building units, but instead dominated by the conformational supramolecular chirality corresponding to different aggregation modes. This chiral-to-chiral communication between dyads introduces multiple stacking preferences, leading to different helical handedness excess within copolymers of the same chiral configuration.

Overall, PICSA serves as a facile and efficient strategy for chiral copolymer self-assembly and *in situ* chiral communication, offering significant advantages in constructing chiroptically active polymer assemblies. The structure–property relationships inherent to PICSA systems enable precise control over supramolecular helical asymmetric structures through modulation of architecture of building units and the DP of the solvophobic block, thereby facilitating programmable switching of chiroptical activity in assembled polymer materials.

#### Chiral communication and transfer in achiral polymers

In achiral polymer systems, the generation of chiroptical activity typically requires the participation of external chiral guest molecules. This strategy bypasses tedious chiral monomer synthesis and minimizes the consumption of expensive chiral reagents. A central approach to inducing helical asymmetry in achiral polymers through chiral communication lies in the efficient transfer of chiral information from the guest molecules to the achiral polymer host, thereby imprinting molecular chirality into the polymer system.

Yashima *et al.* pioneered this field through their seminal contributions to chiral induction and the construction of helical polymers.^64–66^ In 1999, they first reported that chiral amine guest molecules could induce a single-handed helical conformation in poly(phenylacetylene) hosts.^64^ Remarkably, the helical chirality was effectively retained even after the chiral amines were subsequently replaced by achiral ones, demonstrating a remarkable helicity memory effect. Building on this discovery, Yashima *et al.* optimized the polymer structures and successively proposed the concepts of second-generation and third-generation helical chiral memory polymers. During chiral communication and asymmetric amplification, polymer chains undergo folding to form single-handed helical conformations, exhibiting exceptional chiral memory capability. This unique property has led to the successful application of these polymers in enantioseparation.

**Fig. 6:**
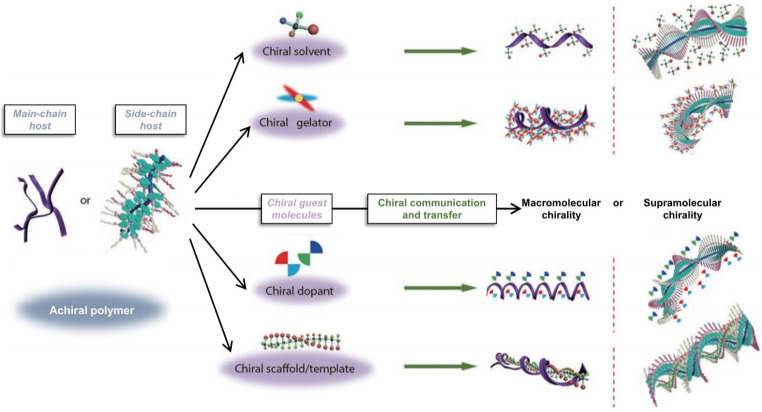
Different chiral induction approaches: chiral solvent induction, chiral gelator induction, chiral dopant induction, and chiral scaffold or template induction; reproduced from ref. 72. Copyright 2021, Springer Nature Limited.

Constructing ordered chiral supramolecular asymmetric structures *via* self-assembly of achiral polymers, wherein a small fraction of chiral guest molecules acts as a “chiral source” to induce chirality in a large number of achiral polymer hosts, has become a widely adopted strategy for developing chiroptical polymer materials.^67–71^ In recent years, a variety of chiral induction approaches have been established, including chiral solvent, chiral gelators, chiral dopants, chiral scaffolds or template induction, *etc.* (Fig. 6).^72^ These strategies have been comprehensively summarized in several excellent reviews and will not be discussed in detail here.

In most cases, chiral information transfer and communication are mediated by the cooperative action of multiple noncovalent interactions. Because these interactions are inherently reversible, the resulting supramolecular structures exhibit dynamic reversibility and high tunability. This, in turn, endows chiral-induced optically active polymer materials with rich structural diversity and versatile functionalities.

## Application prospects

As essential and multi-scale structural building units in nature, chiral helices have motivated extensive biomimetic efforts to recreate their complexity for probing the origins of living life and engineering smart responsive materials. During the chiral communication process, chiral information can undergo multi-level, hierarchical transmission, leading to the formation of corresponding functional chiral architectures. Given this context, this section will briefly outline the potential applications of chiroptical polymers based on both conventional and novel chiral communication strategies.

### Asymmetric catalysis

In asymmetric catalysis, chiral polymers serve not only as supports that enable catalyst recovery and reuse, but also as structural scaffolds that create well-defined chiral microenvironments to regulate enantioselectivity.^73^ This approach provides an attractive pathway for achieving greener and more sustainable asymmetric synthesis. Within this framework, chiral communication plays a pivotal role, both in the synthesis of the chiral polymers themselves and during the asymmetric catalytic process.^74^

**Fig. 7:**
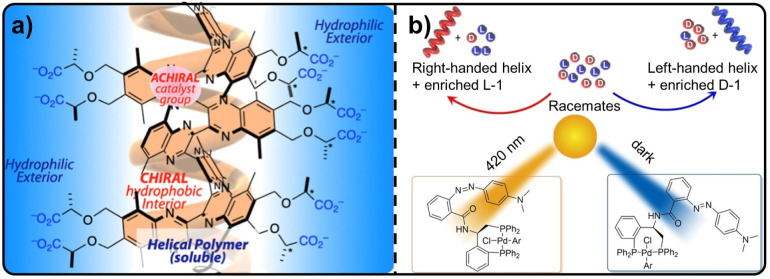
(a) Water-soluble chiral polymer ligand containing catalyst groups for asymmetric catalysis in water; reproduced from ref. 75. Copyright 2025, The American Chemical Society. (b) Photoswitchable enantioselective and helix-sense controlled polymerization of an Azo-modified catalyst; reproduced from ref. 76. Copyright 2023, WILEY-VCH Verlag GmbH & Co. KGaA.

Embedding catalytic groups into helical polymer architectures enables efficient transfer of chiral information, allowing helicity-induced asymmetric catalysis with high enantioselectivity while retaining excellent catalytic activity and stereoselectivity over multiple reuse cycles. Compared with conventional asymmetric catalysis in organic media, the use of water as a more environmentally benign solvent still presents significant challenges, primarily because the hydrophobic nature of many substrates and catalysts hampers both organic reactions and effective chiral information transfer. To address this issue, Suginome, Yamamoto, and co-workers designed and synthesized helical poly(quinoxaline-2,3-diyl) (PQX) bearing chiral carboxylic acid side chains derived from natural *l*-lactic acid. The resulting helical polymers become soluble in alkaline aqueous through the formation of polycarboxylates and adopt a *P*-helix conformation, whereas under weakly acidic aqueous conditions the *M*-helix conformation predominates, enabling controllable switching of helicity (Fig. 7a).^75^ Furthermore, this single-handed helical polymer acts as a ligand for palladium in the Suzuki–Miyaura coupling reaction in water. By effectively transmitting the helical information of the backbone, the system achieves an enantioselectivity as high as the 99% ee value.

Moreover, introducing stimulus-responsive units into catalytic systems allows for the controlled transfer of chiral information under external environmental stimuli, thereby enabling the modulation of catalytic performance. For example, in the Azo-modified catalytic system, owing to the photoinduced *trans*–*cis* isomerization of the Azo unit incorporated into the ligand of the catalyst, the catalytic system exhibits distinct polymerization activity, helical selectivity, and enantioselectivity under irradiation with different wavelengths of light. Under dark conditions, the catalyst enables the efficient synthesis of single-handed helical polymers *via* living polymerization of racemic monomers, while also displaying excellent enantioselectivity toward *L*/*D* enantiomers (Fig. 7b).^76^ In such Azo-based catalytic systems, the sensitivity of the stimulus-responsive group to light irradiation allows for the precise regulation of chiral communication. This provides a mechanism for the light-controlled switching of catalytic effects, paving the way for smart, responsive asymmetric synthesis.

### Chiroptical responsive materials

Artificially synthesized helical polymer materials exhibit excellent processability and thermal stability. At the same time, owing to the versatility of chiral communication mechanisms, their internal asymmetric helical structures are highly tunable. These advantages have led to their widespread applications in chiroptically responsive materials, most notably chiroptical switches.^77–79^

Chiroptical switches refer to materials whose asymmetric structure can undergo reversible changes under external stimuli. Because of the broad potential in optical communication, sensing, bioimaging, and three-dimensional displays, these materials have attracted considerable attention. Supramolecular helical structures formed from achiral Azo building units *via* chiral induction are widely employed for constructing chiroptical switches. However, in achiral polymer systems, once the chiral guest molecules are removed, it is often difficult to recover the initial asymmetric helical arrangement. This limitation originates from the weak and reversible properties of noncovalent interactions, which serve as the primary driving forces for supramolecular chiral self-assembly. Such weak interactions are susceptible to irreversible disruption by external stimuli, and the loss of chiral information from the inducing source consequently leads to permanent disappearance of chiroptical activity.

**Fig. 8:**
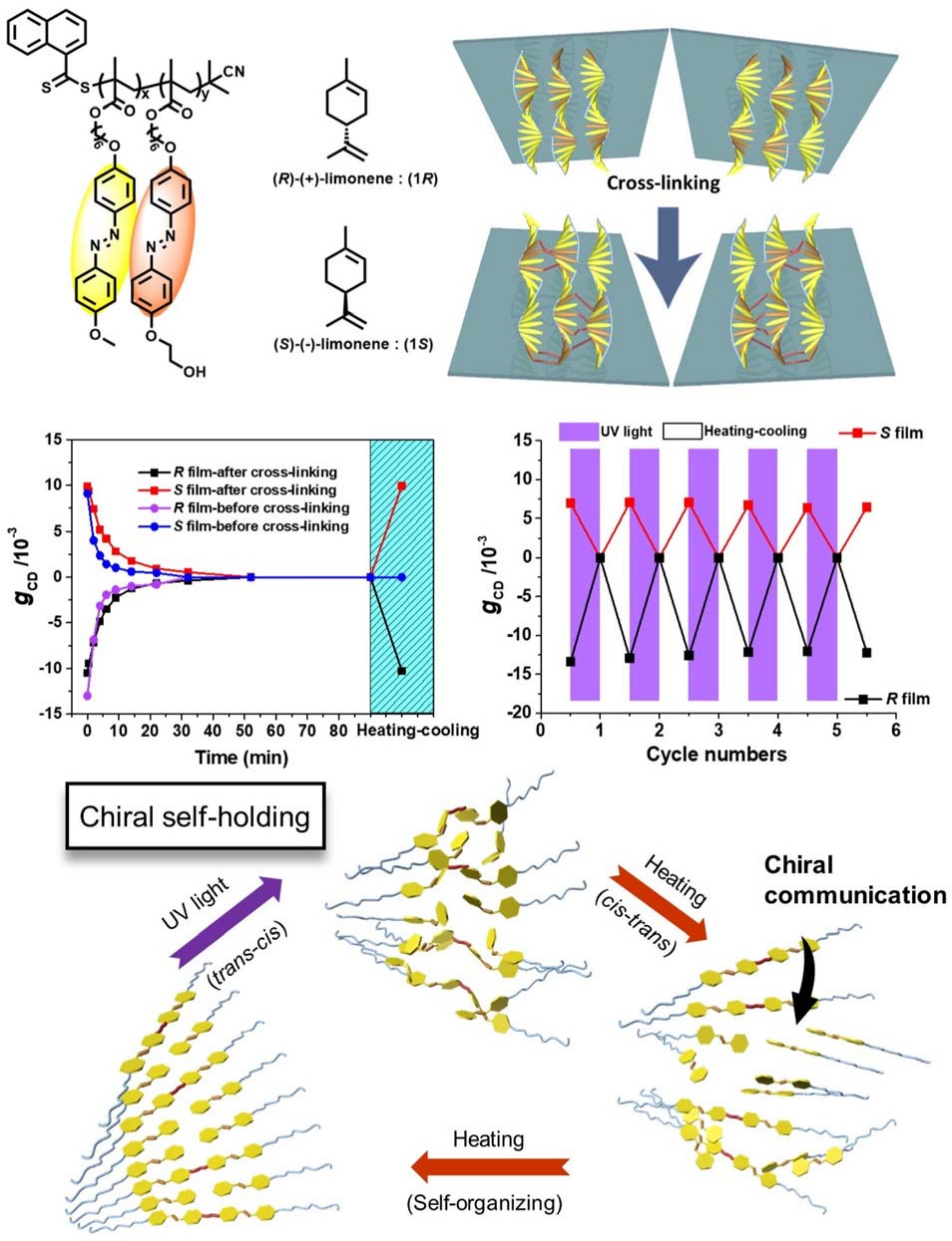
Chiroptical switches in the achiral side-chain polymer and the chiral self-holding strategy; reproduced from ref. 80. Copyright 2021, WILEY-VCH Verlag GmbH & Co. KGaA.

To further improve the stability and tunability of supramolecular asymmetric structures in chiroptical responsive materials, our group has developed a chiral self-holding strategy.^80^ Using an achiral side-chain Azo LC polymer film as the host, we introduce chiral limonene guest molecules *via* vapor induction and then perform covalent crosslinking between the polymer chains. In this way, the chiral information of the guest molecules is transferred and permanently stored in the achiral polymer system. The resulting chiroptical films, endowed with chiral self-holding capability, can undergo complete but temporary loss of chiroptical activity upon thermal, light, or solvent stimulation, as evidenced by the disappearance of CD signals. Remarkably, nearly quantitative recovery of chirality is achieved simply through a heating–cooling cycle, and repeated cycling does not result in noticeable chiral fatigue. In contrast, non-crosslinked chiral polymer films lose their chirality irreversibly under identical experimental conditions (Fig. 8). These results demonstrate that covalent fixation dramatically enhances the chiral memory effect. This strategy, equally applicable to various nano-aggregates,^81^ highlights the broader potential of microscale chiral self-holding concepts in the development of advanced chiroptical responsive materials.

**Fig. 9:**
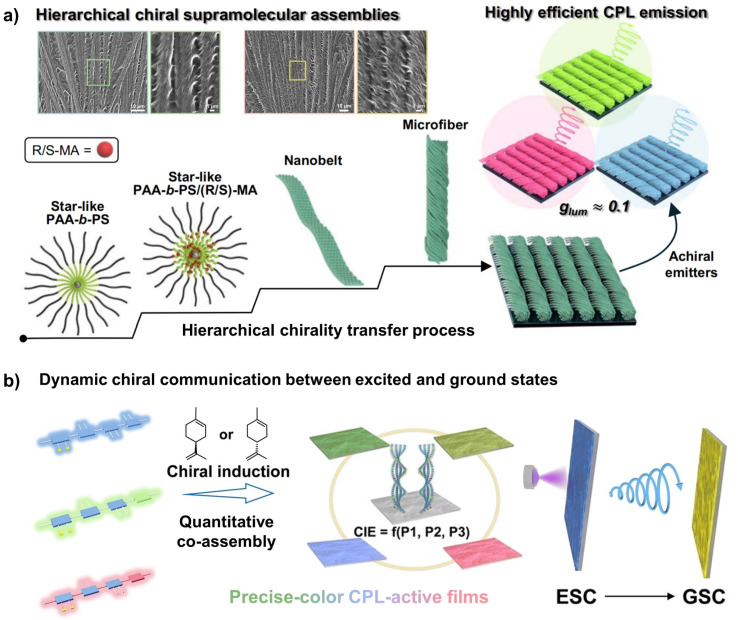
(a) Cooperative self-assembly of the star-shaped block copolymer for creating full-colour, highly efficient CPL-active polymer materials; reproduced from ref. 84. Copyright 2025, The American Association for the Advancement of Science. (b) Dynamic chiral communication between excited and ground states in chiral polymer films governed by a subtraction principle; reproduced from ref. 87. Copyright 2024, WILEY-VCH Verlag GmbH & Co. KGaA.

Beyond absorption properties of chiral materials, chiroptical activities such as CPL have also become a focal point. Supramolecular assembly is a standard approach for fabricating CPL materials, where the emission wavelength can be modulated *via* interactions between chiral donors and achiral fluorescent acceptors.^82^ We prepared supramolecular gels through the co-assembly of polyfluorene derivatives and chiral limonene. By adding achiral fluorophores, we successfully achieved green CPL emission *via* chiral fluorescence resonance energy transfer (C-FRET).^83^ The construction of polymer materials with chiroptical responsiveness often involves a hierarchical chirality transfer process. Recently, Lin and co-workers employed cooperative self-assembly of a star-shaped block copolymer with chiral mandelic acid to construct hierarchical and structurally precise chiral supramolecular materials.^84^ In this system, the chiral small molecules transfer the chiral information to poly(acrylic acid) chains *via* hydrogen bonding, followed by further stacking of the star polymers into transient nanoribbons. Over time, these nanoribbons hierarchically assemble into stable and micron-scale chiral fibrous rope-like structures. By integrating multifarious achiral luminescent molecules and nanocrystals into this chiral supramolecular system, stable and reversible CPL with high dissymmetry factors was achieved across multiple colours (Fig. 9a).

Moreover, an alternative and highly effective strategy for constructing CPL-active helical polymers involves the direct incorporation of emissive units into the polymer backbone *via* copolymerization.^85^ The fluorophores can be embedded in the polymer backbone or attached to the side-chains. Deng and co-workers introduced achiral pyrene groups into the side-chains of helical polyacetylenes.^86^ The resulting helical polymers, formed through chiral communication, exhibited opposite CPL signals in solution and solid-state films, with |*g*_lum_| values in the film state exceeding those in solution by more than two orders of magnitude. They further proposed a matching rule for CPL in thin films, providing a new paradigm for constructing tunable CPL materials with exceptionally high |*g*_lum_| values. Recent studies have shown that applying this matching rule to ordered combinations of achiral fluorescent films and chiroptical Azo polymer films can not only achieve full-colour CPL emission from violet to red and white and room-temperature phosphorescent CPL, but can also be used to construct CPL-based optical switches. Such universal and switchable CPL platforms hold great promise for excited-state chiral information storage and encryption.

In addition, our group recently proposed a novel chiral communication strategy that enables dynamic and reversible modulation of CPL. By constructing a dual-film system composed of designed polyfluorene derivatives and chiroptical side-chain Azo polymers, precise control over CPL attenuation, inversion, and enhancement was achieved through dynamic chiral communication between excited and ground states, governed by a subtraction principle (Fig. 9b).^87^ Building on this chiral communication process, we further demonstrated a higher-order dynamically switchable QR code, providing a new and versatile strategy for the precise construction and dynamic regulation of CPL-active materials.

### Biomimetic materials

Asymmetric structures are fundamental building units in nature, spanning a wide range of length scales. From the biomimetic perspective, researchers have long sought to emulate the complex shape transformations and motion modes observed in natural and living systems, with the aim of elucidating the origins of life and developing intelligent and stimuli-responsive biomimetic materials.^88,89^

In the natural world, chiral structural colour is ubiquitous. For instance, the metallic luster of beetle shells originates from such structures, providing unique optical information in the state of polarized light that significantly enhances their survival.^90^ Such naturally occurring chiral structural colours have inspired extensive research across diverse scientific disciplines. Cellulose nanocrystals (CNCs), extracted from natural cellulose, serve as an essential material for fabricating iridescent LC self-assembled films with superior circularly polarized light properties. From a supramolecular perspective, comparable optical effects can also be achieved by inducing supramolecular chiral self-assembly in synthetically accessible achiral polymers *via* chiral information transfer, while simultaneously endowing the resulting materials with enhanced and tunable functionalities.

**Fig. 10:**
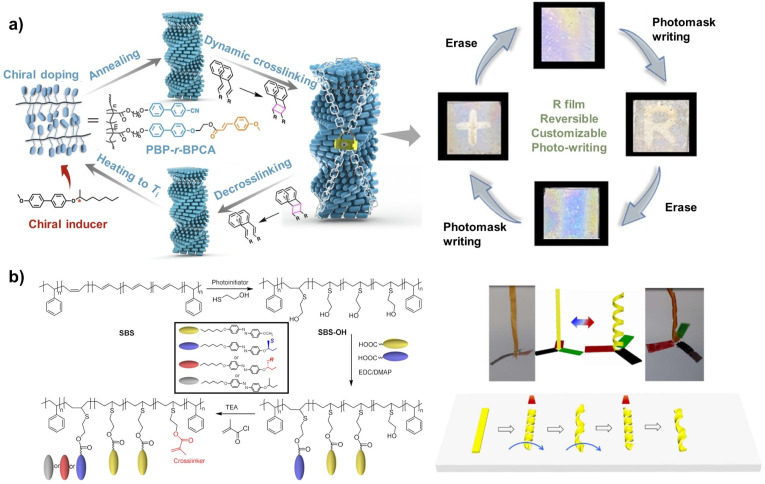
(a) Dynamic chiral fixation process and storage mechanism in chiral polymer superstructures and the reversible anti-counterfeiting characteristics with information encryption of an embedded pattern; reproduced from ref. 91. Copyright 2023, WILEY-VCH Verlag GmbH & Co. KGaA. (b) Synthetic route of the LC elastomers (LCEs) bearing Azo mesogens and the unconventional macroscopic helical behaviours of multidomain LCE films; reproduced from ref. 93. Copyright 2021, The American Chemical Society.

For instance, achiral side-chain LC polymers can serve as the host, into which photoresponsive cinnamate groups are incorporated as reversible crosslinking sites. After the introduction of chiral information through induction by chiral guest molecules, photo-regulated reversible [2 + 2] cycloaddition of the cinnamate units enables controlled inter-chain crosslinking and decrosslinking, thereby allowing the supramolecular chiral structures to be “locked” and “unlocked.” Benefiting from the ordered helical arrangement within the LCP, structural colour patterns can be precisely drawn through appropriate annealing and localized crosslinking. Meanwhile, upon disruption of the internal helical order, subsequent annealing enables the transfer of chiral information from the crosslinked regions to reconstruct the ordered helical structure of the structural colour film, thereby realizing erasable and rewritable patterned information (Fig. 10a).^91^ This strategy enables reversible, embedded pattern storage and readout in chiral LC polymer systems.

Although numerous elegant studies have reported chiral helical structures across microscopic and macroscopic length scales, the direct fabrication of dynamically responsive materials capable of helical shape transformation, particularly planar two-dimensional to three-dimensional helical transitions, remains highly challenging. This difficulty arises from the inherently multiscale nature of chiral information transfer, in which higher-order asymmetric structures (*e.g.*, macroscopic helical chirality) emerge from complex interactions among lower-level chiral elements, rendering the formation process considerably more intricate.^92^ In recent years, the preparation and regulation of macroscopic helical LC elastomers (LCEs) have attracted increasing attention. Under external stimuli such as light or heat, artificial helical materials can undergo programmable shape changes or motions, demonstrating substantial potential in precision optomechanical devices, adaptive robotics, and sustainable energy harvesting.

Typically, chiral small molecules are doped into polymer LCE films, where chiral information can be transferred from the chiral molecules to the LC polymer network, enabling the fabrication of helical ribbons with tunable pitch and handedness through mechanical shearing.^7^ Notably, conventional LCE processes generally require monodomain LC alignment to achieve stimuli-responsive helical deformation. In contrast, when chiral Azo small molecules, achiral Azo mesogens, and crosslinking sites are respectively grafted onto the polybutadiene chains of a styrene-butadiene-styrene (SBS) block copolymer, multidomain LCE films can be readily prepared *via* solution casting and thermal annealing. In this system, chiral information undergoes multilevel transfer and asymmetry amplification, ultimately forming macroscopic helical splines. Such multidomain chiral LCE films exhibit unconventional macroscopic helical behaviours and offer simpler and more accessible routes to mimicking plant tendril-like helical deformations, achieving continuous helical rolling motion, and performing mechanical work (Fig. 10b).^93^ These insights into macroscopic chirality provide a new paradigm for the development of next-generation chiral liquid crystalline actuators.

## Conclusions and perspective

Over the past few decades, helical polymers with tunable chiroptical activity have gained increasing recognition due to their potential applications in asymmetric catalysis, chiral nanoreactors, and optoelectronic devices. Chiral communication, as the central mechanism governing the generation, amplification, and regulation of asymmetric structures in polymer systems, has recently emerged as a powerful tool for understanding and constructing chiral polymer materials. Through the effective transfer and coupling of chiral information across molecular, supramolecular, and even macroscopic length scales, polymer systems can exhibit complex chiral functions that far exceed those dictated by the intrinsic stereochemical features. Meanwhile, the use of chiral communication among building units in polymers has emerged as a highly efficient and versatile strategy for regulating asymmetric helical architectures. This approach enables the amplification of molecular level asymmetry into well-defined nano- and even micro-scale ordered structures, thereby endowing the resulting materials with unique properties.

With rapid advances in supramolecular chemistry, a growing number of asymmetric amplification strategies, such as the recently reported “helicity overriding” behaviour,^94^ have been developed in the field of supramolecular chiral self-assembly. However, the introduction of novel chiral communication pathways into polymer systems remains challenging due to the complex interplay of intra-chain and inter-chain interactions among the building units. However, this multiplicity of interactions also enhances structural stability, presenting new opportunities for the design of chiral polymer materials. Therefore, achieving dynamic and precise control over chiral helical structures through tailored chiral communication, along with developing novel chiral transfer strategies, represents a key frontier in the ongoing research on chiral polymers. Although significant advances have been made in recent years, systematic reviews that specifically address chiral communication mechanisms in polymer systems remain limited. This perspective attempts to fill this gap by focusing on how chirality is communicated and transferred across multiple hierarchical levels in polymer asymmetric structures.

Furthermore, the competition and cooperation among different chiral building units, the transition between kinetic and thermodynamic control during the assembly process, and the influence of phase transitions on chiral evolution often result in highly complex behaviours in polymer systems. Meanwhile, the intrinsic polydispersity of polymers further complicates the chiral communication processes among the constituent building units and the pathways of hierarchical assembly. Future progress will require the integration of advanced *in situ* characterization techniques with theoretical modelling to elucidate the fundamental principles governing the hierarchical transfer of chirality, thereby laying a solid foundation for the development and application of next-generation intelligent chiroptical polymer materials.

## Author contributions

Z. H. and W. Z. conceived the focus of the perspective. Z. H. and G. Z. performed the literature search, analysed the published results, and wrote the manuscript. W. Z. revised and supervised the manuscript.

## Conflicts of interest

There are no conflicts to declare.

## Data Availability

No primary research results, software or code have been included and no new data were generated or analysed as part of this perspective paper.
